# *SRY*-Related Transcription Factors in Head and Neck Squamous Cell Carcinomas: In Silico Based Analysis

**DOI:** 10.3390/cimb45120592

**Published:** 2023-11-24

**Authors:** Tomasz Kolenda, Zuzanna Graczyk, Barbara Żarska, Wojciech Łosiewski, Mikołaj Smolibowski, Adrian Wartecki, Joanna Kozłowska-Masłoń, Kacper Guglas, Anna Florczak, Urszula Kazimierczak, Anna Teresiak, Katarzyna Lamperska

**Affiliations:** 1 Laboratory of Cancer Genetics, Greater Poland Cancer Centre, Garbary 15, 61-866 Poznan, Poland; 2 Research and Implementation Unit, Greater Poland Cancer Centre, Garbary 15, 61-866 Poznan, Poland; 3 Department of Cancer Immunology, Poznan University of Medical Sciences, 8 Rokietnicka Street, 60-806 Poznan, Polanda.wartecki25@gmail.com (A.W.); annaflorczak@ump.edu.pl (A.F.);; 4 Institute of Human Genetics, Polish Academy of Sciences, Strzeszynska 32, 60-479 Poznan, Poland; 5 Institute of Human Biology and Evolution, Faculty of Biology, Adam Mickiewicz University, Uniwersytetu Poznańskiego 6, 61-614 Poznan, Poland; 6 Postgraduate School of Molecular Medicine, Medical University of Warsaw, Żwirki i Wigury 61, 02-091 Warsaw, Poland; 7 Department of Diagnostics and Cancer Immunology, Greater Poland Cancer Centre, Garbary 15, 61-688 Poznan, Poland

**Keywords:** *SOX2-OT*, *SOX6*, *SOX8*, *SOX21*, *SOX30*, *SRY*, HNSCC, long non-coding RNA, miRNA, biomarker, TCGA, *SRY*-related transcription factors

## Abstract

Head and neck squamous cell carcinoma (HNSCC) is the sixth leading cancer and the fifth cause of cancer-related deaths worldwide with a poor 5-year survival. *SOX* family genes play a role in the processes involved in cancer development such as epithelial–mesenchymal transition (EMT), the maintenance of cancer stem cells (CSCs) and the regulation of drug resistance. We analyzed the expression of *SOX2-OT*, *SOX6*, *SOX8*, *SOX21*, *SOX30* and *SRY* genes in HNSCC patients using the Cancer Genome Atlas (TCGA) and Gene Expression Omnibus (GEO) datasets, to assess their biological role and their potential utility as biomarkers. We demonstrated statistically significant differences in expression between normal and primary tumor tissues for *SOX6*, *SOX8*, *SOX21* and *SOX30* genes and pointed to *SOX6* as the one that met the independent diagnostic markers criteria. *SOX21* or *SRY* alone, or the panel of six *SRY*-related genes, could be used to estimate patient survival. *SRY*-related genes are positively correlated with immunological processes, as well as with keratinization and formation of the cornified envelope, and negatively correlated with DNA repair and response to stress. Moreover, except *SRY*, all analyzed genes were associated with a different tumor composition and immunological profiles. Based on validation results, the expression of *SOX30* is higher in HPV(+) patients and is associated with patients’ survival. *SRY*-related transcription factors have vast importance in HNSCC biology. *SOX30* seems to be a potential biomarker of HPV infection and could be used as a prognostic marker, but further research is required to fully understand the role of *SOX* family genes in HNSCC.

## 1. Introduction

Head and neck squamous cell carcinoma (HNSCC) is localized in the oral cavity, oropharynx, hypopharynx and nasopharynx [1,2,3]. The most important risk factors are tobacco use, alcohol consumption and human papillomavirus (HPV) infection [3,4]. HNSCC is the sixth leading cancer and the fifth cause of cancer-related deaths worldwide [3,4], with 600,000 new cases every year. HNSCC has a poor 5-year survival (40–60%), causing 2% of all deaths in men (data for larynx cancers) in the Greater Poland Region [1,2]. Treatment is based on surgery, radiation and chemotherapy [1]. HNSCC can be divided into two main groups, HPV-positive and HPV-negative, and such a status constitutes a significant prognostic factor. HPV(+) cancers are mainly caused by oral sex and mainly located in the oropharynx and HPV(−) cancers are mostly caused by smoking and excessive alcohol use and they have different molecular features [4,5,6,7].

Based on gene expression profiles, the four subtypes of HNSCC have been distinguished: basal (31%), mesenchymal (27%), atypical (24%) and classical (18% of cases). They differ clinically, histologically and molecularly. Genetic heterogeneity results in the activation of protein-coding oncogenes, e.g., *EGFR* and *PIK3CA*, and a loss of function in tumor-suppressor genes, e.g., *p53* and *p16* [4]. In spite of the identification of the genes involved in the development and prognosis of HNSCC [8,9], no dramatic changes in recovery rates have been observed. However, these protein-coding transcripts constitute only about 2% of the human genome, and most of the genome consists of the non-coding genes which are responsible for regulation of cellular processes [10,11,12]. Among them, we can distinguish long non-coding RNAs (lncRNAs) which are about 200 nucleotide-length transcripts constituting more than 80% of the eukaryotic transcriptome [11]. Many lncRNAs are dysregulated in some cancers and can be used as biomarkers [13,14,15,16]. lncRNAs participate in stem cell differentiation by regulating the pluripotency and are involved in tumorigenesis as tumor oncogenes or suppressors regulating cancer-related signaling pathways [17].

The *SOX* family is the *SRY*-related transcription factor family and includes 30 members [18]. These proteins are involved in several growth and development processes, e.g., embryonic development, sex determination, stem cell formation and angiogenesis [19]. *SOX* genes also have a role in cancer development including important cancer-related processes such as epithelial–mesenchymal transition (EMT) and the maintenance of cancer stem cells (CSCs) population, as well as the regulation of drug resistance [20]. Moreover, long non-coding RNAs associated with the *SOX* gene, such as *SOX2* overlapping transcript (*SOX2-OT*), are dysregulated in various tumors and function as oncogenes [21]. The role of *SRY*-related transcription factors and their non-coding-related transcripts is not fully understood in the case of HNSCC. In spite of many different strategies for improving the diagnosis [22,23,24] and chemo- [25] and/or radiotherapy treatment of HNSCC patients, an improvement in patients’ outcomes is still challenging [26,27,28]. Probably, treatment personalization based on a molecular transcription profile including both coding and non-coding genes could help in overcoming the high mortality rate.

In this study, the selected *SRY*-related transcription factors, *SOX2-OT*, *SOX6*, *SOX8*, *SOX21*, *SOX30* and *SRY*, were analyzed, and their biological role and their potential utility as biomarkers for HNSCC were tested.

## 2. Materials and Methods

### 2.1. TGCA Data

The TCGA expression and clinical data of *SOX2-OT*, *SOX6*, *SOX8*, *SOX21*, *SOX30* and *SRY* were downloaded from cBioPortal and from the Santa Cruz University of California dataset (Head and Neck Squamous Cell Carcinoma, TCGA, dataset: gene expression RNAseq—IlluminaHiSeq pancan-normalized; RNA expression pan-cancer-normalized log2(norm_count + 1); accessed on 1 November 2021 from https://xenabrowser.net/) and from the UALCAN databases (http://ualcan.path.uab.edu; accessed on 1 November 2021) [29,30]. All data are available online; access is unrestricted and does not require patient’s consent or other permissions. The use of the data does not violate the rights of any person or any institution.

### 2.2. Data Analysis

The expression levels of *SOX2-OT*, *SOX6*, *SOX8*, *SOX21*, *SOX30* and *SRY* genes were analyzed and correlated with clinicopathological parameters: age (<61 vs. >61), smoking category (No vs. Yes and Ex-smoker), gender (women vs. men), alcohol history (negative vs. positive), cancer stage (I + II vs. III + IV), T-stage (T1 + T2 vs. T3 + T4), N-stage (N0 + N1 vs. N2 + N3), grade (G1 + G2 vs. G3 + G4), perineural invasion (negative vs. positive), lymph node neck dissection (negative vs. positive), lymphovascular invasion (negative vs. positive) and HPV in p16 test (negative vs. positive) in all localizations of the HNSCC samples as described previously [31,32]. The GEPIA2 tool was used to estimate the survival rates for patients taken for the TCGA using the mean of the *SRY*-related genes expression as the cut-off.

### 2.3. Gene Analysis

Association between *SOX2-OT*, *SOX6*, *SOX8*, *SOX21*, *SOX30* and *SRY* expression levels was analyzed using the UALCAN database. Receiver operating characteristic (ROC) analysis with an area under the curve (AUC) estimation in a group of 43 adjacent-matched healthy and neoplastic tissues was applied to determine the utility of diagnostic markers for distinguishing healthy from cancer samples. Next, a correlation between expression levels of *SOX2-OT*, *SOX6*, *SOX8*, *SOX21*, *SOX30* and *SRY* was obtained using the Spearman test (positive correlation: r > 0.3; negative correlation: r < −0.3) and a heatmap was created using Morpheus Software (https://software.broadinstitute.org/morpheus; accessed on 1 March 2021). Next, expression levels of *SRY*-related genes were checked depending on localizations in the oral cavity (*n* = 346), pharynx (*n* = 88) and larynx (*n* = 130) according to National Institutes of Health (NIH) classification.

### 2.4. Association of SRY-Related Transcripts in Context to Stromal and Immune Scores and Immune Profile

Tumor composition scores for this analysis were acquired from the ESTIMATE (https://bioinformatics.mdanderson.org/public-software/estimate/; accessed on 2 January 2021) database. Data from ESTIMATE contains information about the presence of stroma in tumor tissues (stromal score), representation inflation of immune cells in tumor tissues (immune score) and tumor purity (estimate score). We separated each gene expression group in two, low and high relative to average in all samples, as described previously [30,31,32,33,34].

### 2.5. Validation of the Results

We used the Gene Expression Omnibus (GEO) data repository, with GSE65858 [35] and GSE3292 [36] datasets to validate our results for *SOX2-OT*, *SOX6*, *SOX8*, *SOX21*, *SOX30* and *SRY* genes. The expression levels of *SRY*-related transcripts were compared between HPV(−) and HPV(+), types of HPV and between an active infection and inactive infection. Next, we evaluated the differences between the expression levels of *SRY*-related transcripts and various clinicopathological parameters such as age, gender, smoking history, alcohol consumption, disease stage, T stage, N stage, cancer molecular clusters types and tumor localization. Next, we analyzed patients’ progression-free interval (PFI) and overall survival (OS) in all patients, only in the HPV(+) and only in the HPV(−) groups of patients, using the mean of expression in analyzed groups as a cut-off (low vs. high). The validation of the results was performed similarly to described previously [30]. The statistical analyses were performed as described in point 2.6.

### 2.6. Statistical Analysis

Statistical analyses were performed using GraphPad Prism 5/8 (GraphPad, San Diego, CA, USA). The Shapiro–Wilk normality test and Mann–Whitney U test were used for measuring *SOX2-OT*, *SOX6*, *SOX8*, *SOX21*, *SOX30* and *SRY* levels (depending on clinical parameters). The expression in each of *SOX2-OT*, *SOX6*, *SOX8*, *SOX21*, *SOX30* and *SRY* genes was compared depending on the location of the tumor using one-way ANOVA, obtained using the Tukey test. All TCGA data are presented as a median with SEM (standard error of the mean). Disease-free survival (PFS) and OS survival rates and statistics for data from the TCGA were generated using GEPIA2 tool. PFI and OS for data from GEO datasets were calculated using the log-rank (Mantel–Cox) test and the Gehan–Breslow–Wilcoxon test. In all analyses, *p* < 0.05 was used to determine statistical significance, as described previously [30,31,32,33,34].

## 3. Results

### 3.1. SOX Family Gene Expression Levels Distinguish between Healthy and Cancer Tissues

**Figure 1:**
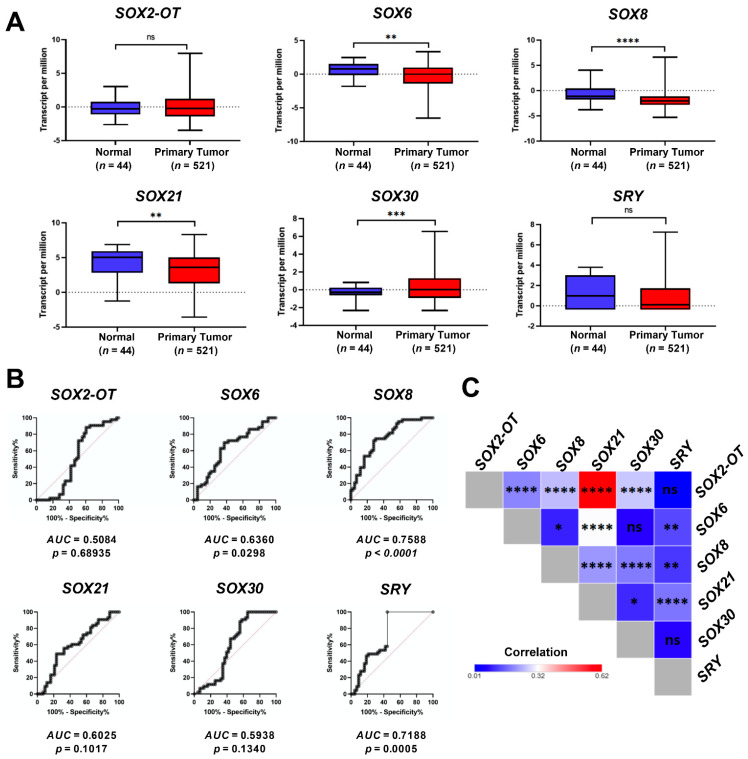
The expression level of *SOX2-OT*, *SOX6*, *SOX8*, *SOX21*, *SOX30* and *SRY* in HNSCC patients: (**A**) differences in gene expression between normal (blue boxes) and cancer tissues (red boxes), data from UALCAN presented as log2(TPM + 1), modified; (**B**) receiver operating characteristic curve (ROC) analyses of *SOX2-OT*, *SOX6*, *SOX8*, *SOX21*, *SOX30* and *SRY* expression levels in HNSCC patients; (**C**) the correlation matrix between expression levels of *SRY*-related genes in HNSCC patients; Spearman correlation test with *p* < 0.05 considered as significant; ns: not significant, * *p* < 0.05; ** *p* < 0.01; *** *p* < 0.001; **** *p* < 0.0001.

We observed statistically significant differences in the expression of *SOX6*, *SOX8*, *SOX21* and *SOX30* genes in healthy tissue vs. primary tumor samples. In all cases, except for *SOX30*, the expression of these genes was lower in cancer tissues compared to the healthy ones. No differences between tumor and normal tissues were indicated for *SOX2-OT* and *SRY* genes (*p* > 0.05), Figure 1A.

Next, receiver operating characteristic curve (ROC) analyses for *SOX2-OT*, *SOX6*, *SOX8*, *SOX30* and *SRY* were performed to describe the potency of *SRY*-related genes to distinguish between healthy and cancerous samples. We showed that the analysis of the expression levels of *SOX6*, *SOX8* and *SRY* correlated with the AUC (area under the curve) estimated to 0.6360, 0.7588 and 0.7188, respectively, *p* < 0.05. *SOX2-OT*, *SOX21* and *SOX30* did not meet the criteria of the diagnostic markers (*p* > 0.05, AUC < 0.6), Figure 1B.

To analyze the correlation between all *SRY*-related genes in HNSCC samples, the Spearman test was used. The results showed that *SOX2-OT* expression was positively correlated with *SOX21* (R = 0.6153, *p* < 0.0001), and also, *SOX6* expression was positively correlated with *SOX21* (R = 0.3130, *p* < 0.0001). No correlations (*p* > 0.05) between *SOX30* and *SOX6*, *SOX30* and *SRY* or *SRY* and *SOX2-OT* were indicated, Figure 1C.

### 3.2. Expression Levels of SRY-Related Transcription Factors Depend on Tumor Localization

**Figure 2:**
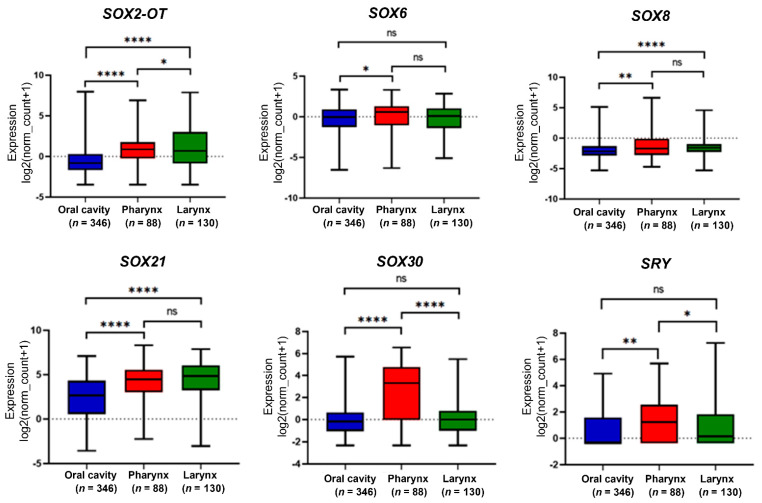
Expression levels of *SOX2-OT*, *SOX6*, *SOX8*, *SOX21*, *SOX30* and *SRY* genes in patients with head and neck squamous cell carcinoma depending on the localization of the tumor. Graphs show the mean value of transcripts per million; *t*-test or Mann–Whitney U test; *p* < 0.05 considered as significant; ns: not significant, * *p* < 0.05; ** *p* < 0.01; **** *p* < 0.0001.

In our analyses, we divided the patients into three groups, oral cavity (*n* = 346), pharynx (*n* = 88) and larynx (*n* = 130), depending on the tumor localization based on the National Institutes of Health (NIH) classification. The gene expression in each group was compared for *SOX2-OT*, *SOX6*, *SOX8*, *SOX21*, *SOX30* and *SRY* genes. For the oral vs. pharynx comparison, there were significant differences between expressions in all genes except *SOX6* (*p* > 0.05). There were no significant differences between the pharynx and larynx in all genes other than *SOX2-OT*, *SOX30* and *SRY*. Between the oral cavity and larynx, there were significant differences in *SOX2-OT*, *SOX8* and *SOX21*, Figure 2.

### 3.3. Expression Levels of SRY-Related Transcription Factors Depend on Specific Clinicopathological Parameters

Differences in expression levels of all studied genes in HNSCC patients were analyzed in the context of selected clinicopathological parameters. In all parameters, the expression of at least one of the six studied genes showed a significant difference. *SOX2-OT*, *SOX6*, *SOX8*, *SOX21* and *SRY* genes were connected with tobacco smoking; *SOX2-OT*, *SOX6*, *SOX21* and *SOX30*- were connected with lymph node neck dissection and lymphovascular invasion; and *SOX2-OT*, *SOX8* and *SOX30* were connected with HPV in p16 test two with regard to gender (*SOX21*, *SRY*), cancer stage (*SOX6*, *SOX21*) and grade (*SOX8*, *SOX30*) and one gene with age (*SRY*), alcohol (*SRY*), T stage (*SOX8*) and N stage (*SOX30*). Data are summarized in detail in Table 1 and Table 2.

### 3.4. Higher Survival Rates Are Associated with High SOX21 and SRY Expression Levels

**Figure 3:**
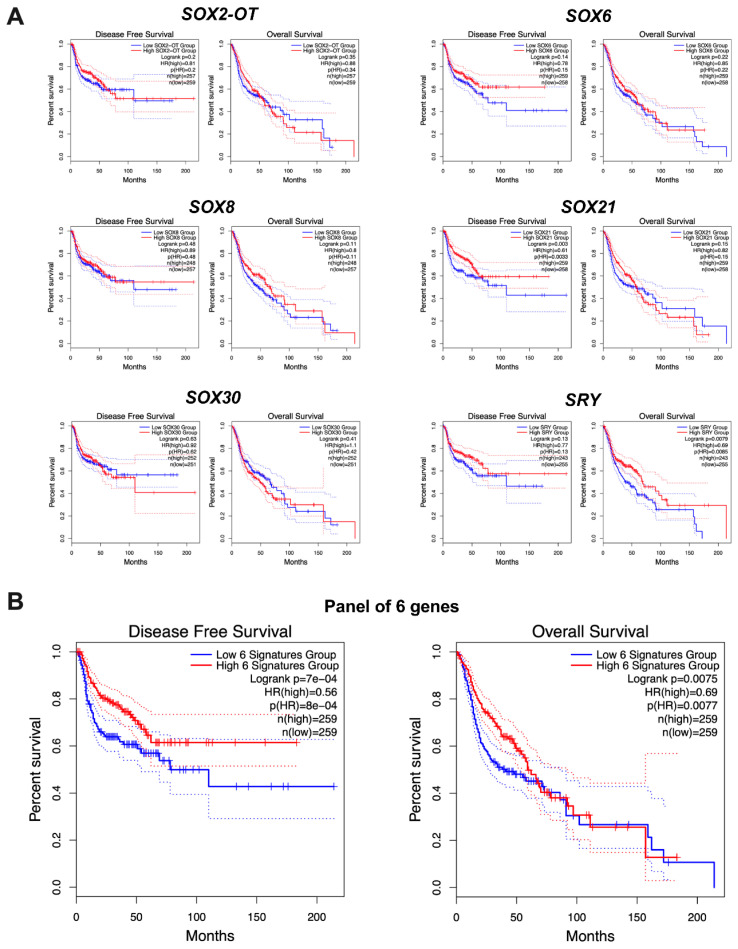
Disease-free survival and overall survival rates of HNSCC patients with low and high expression of each of the transcripts (**A**) separately and (**B**) together. Patients were divided into low and high groups based on the median expression of each gene; graphs generated using GEPIA2 tool; *p* < 0.05 considered as significant.

Patients were divided into two groups with low and high expression levels of the selected genes. The high expression group was distinguished from the low expression group on the basis of the median of all patients gene expression levels. For each gene, both groups had their overall survival and disease-free survival rates compared. There were no significant differences (*p* > 0.05) in any of the analyzed genes, except for disease-free survival in *SOX21* and for overall survival for the *SRY*, which had a better survival rate when the expression of this gene was higher than the median. HNSCC patients with higher levels of the six examined *SRY*-related transcription factors have longer DFS and OS in comparison to the patients with lower levels of these transcripts, Figure 3.

### 3.5. SOX Genes’ Expression Levels Correlate with Important Cellular Processes

**Figure 4:**
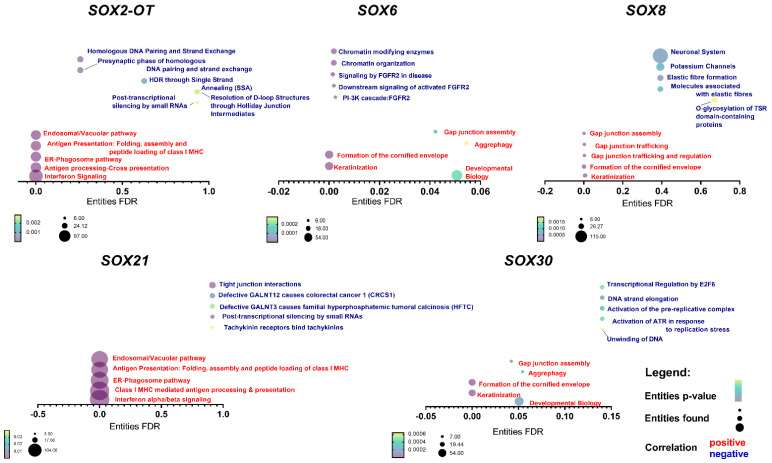
Positive and negative correlation of *SOX6*, *SOX2-OT*, *SOX8*, *SOX21* and *SOX30* with genes involved in the important cellular processes. Only genes with Spearman’s correlation R > 0.3, R < −0.3 and *p* < 0.05 were indicated in the REACTOME pathway analysis. Green color indicates negatively correlated genes, and positively correlated genes are in orange.

Positively and negatively (Spearman positive correlation: R > 0.3; negative correlation: R < −0.3) correlated genes with *SOX2-OT*, *SOX6*, *SOX8*, *SOX21*, *SOX30* and *SRY* were downloaded from cBioPortal.com, accessed on 2 January 2021, see Supplementary Materials. The gene-pathways association analysis was performed using the Reactome web tool reactome.org (accessed on 1 March 2021). Five pathways were selected representing each set with the lowest *p* value. *SRY* was not included in the analysis due to the insufficient number of documented correlations. For *SOX6,* negatively correlated genes were associated with chromatin organization and *FGFR2* signaling, and positively correlated genes were associated with developmental biology processes, keratinization and formation of the cornified envelope. For *SOX8,* processes such as O-glycosylation of the TSR domain-containing proteins, fiber formation and potassium channels were connected with negatively correlated genes, and those which were positively correlated were similar to the *SOX6* with additional processes connected with the gap junction. In the case of *SOX30*, positively correlated genes were also similar to those described for *SOX6* and *SOX8*, but negatively correlated genes were associated with DNA replication processes. The last protein-coding gene, *SOX21*, correlated with genes associated with very different processes in comparison to the rest of *SRY*-related genes. Positively correlated genes were connected with immunological processes and cellular transport, and negatively correlated ones with tight junction interactions and with N-acetylgalactosaminyltransferase (*GALNT3* and *GALNT12*). Finally, our analysis indicated that *SOX2-OT* lncRNA were positively correlated with genes associated with immunological processes and cellular transport, similar to *SOX21*. On the other hand, negatively correlated genes were connected with DNA repair. All data are presented in Figure 4 and in Supplementary Table S1.

### 3.6. Tumor Composition Is Associated with Differences in the Expression of SRY-Related Transcription Factors

Next, we focused on the tumor composition using a stromal score that indicates the presence of stroma in tumor tissue, an immune score describing the infiltration of immune cells in tumor tissue and an estimate score that describes the tumor purity.

**Figure 5:**
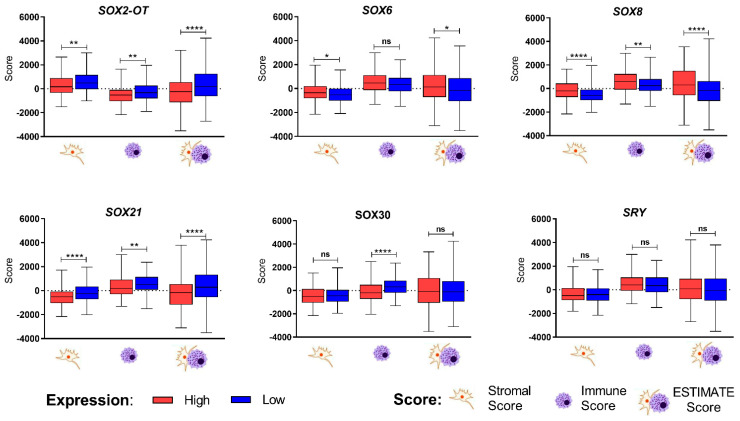
Differences between patients with low and high expression of *SRY*-related genes in context of their stromal score, immune score and estimate score; *t*-test or Mann–Whitney U test; *p* < 0.05 considered as significant; ns: not significant, * *p* < 0.05; ** *p* < 0.01; **** *p* < 0.0001.

We found that the stromal score, immune score and estimate score were lower in patients with a higher expression of *SOX2-OT* and *SOX21* and with a lower expression of *SOX8*. Stromal and estimated scores were higher in samples with lower *SOX6* expression. The immune score was higher in samples with higher *SOX30* expression, Figure 5 and Supplementary Table S2.

**Figure 6:**
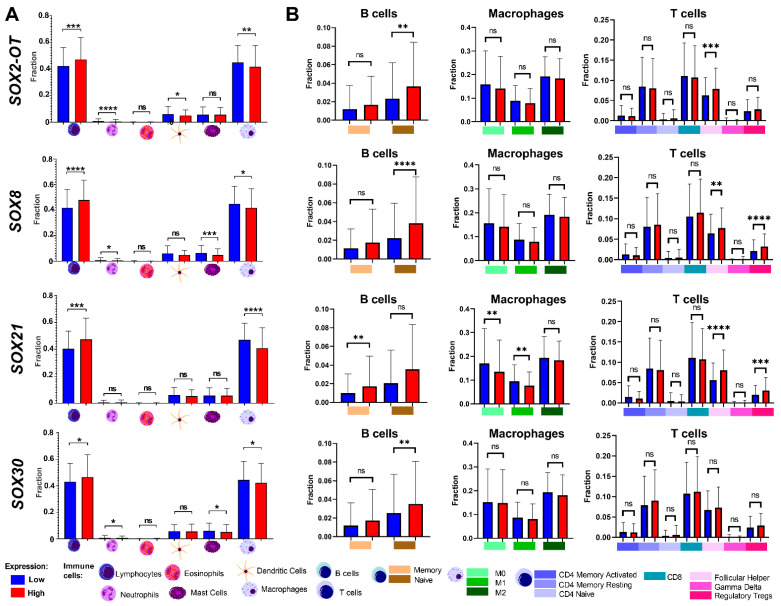
Immunological profile of HSNCC patients depending on the expression levels of *SOX2-OT*, *SOX8*, *SOX21* and *SOX30*. Differences in the population of lymphocytes, neutrophils, eosinophils, mast cells, dendritic cells and macrophages (**A**), and in the specific subpopulation of B cells, macrophages and T cells (**B**). *t*-test or Mann–Whitney U test; *p* < 0.05 considered as significant; ns: not significant, * *p* < 0.05; ** *p* < 0.01; *** *p* < 0.001; **** *p* < 0.0001.

For genes with significant correlations with the immune scores, we analyzed the specific populations of immune cells, Figure 6. We found that in patients with a higher expression of *SOX2-OT,* the population of lymphocytes was significantly elevated, and the populations of neutrophils, mast cells and macrophages were lowered. Similar results were found in *SOX8*, *SOX21* and *SOX30* analyses. The population of neutrophils and dendritic cells were lowered in patients with a higher expression of *SOX8* and *SOX30*. A higher expression of *SOX8*, *SOX21* and *SOX2-OT* correlated with the lowered population of macrophages, Figure 6A and Supplementary Table S3A.

We also analyzed the subpopulations of B cells, macrophages and T cells. The subpopulation of naive B cells was higher in patients with a higher expression of *SOX2-OT*, *SOX8* and *SOX30*, and in patients with a higher expression of *SOX21,* the subpopulation of memory B cells was higher. The significant differences of the subpopulation of macrophages were found in *SOX21*. The subpopulation of M0 and M1 macrophages were lower in patients with a higher expression of this gene. There were no significant differences in subpopulations of T cells in the *SOX30* gene. In *SOX8* and *SOX21,* the subpopulation of follicular helper T cells and regulatory Tregs were higher in patients with a higher expression, and in *SOX2-OT,* the subpopulation of follicular helper T cells was higher in patients with a higher expression. All data are presented in Figure 6B and in Supplementary Table S3B.

### 3.7. Validation of the Results for SRY-Related Transcription Factors Using the GEO Datasets

**Figure 7:**
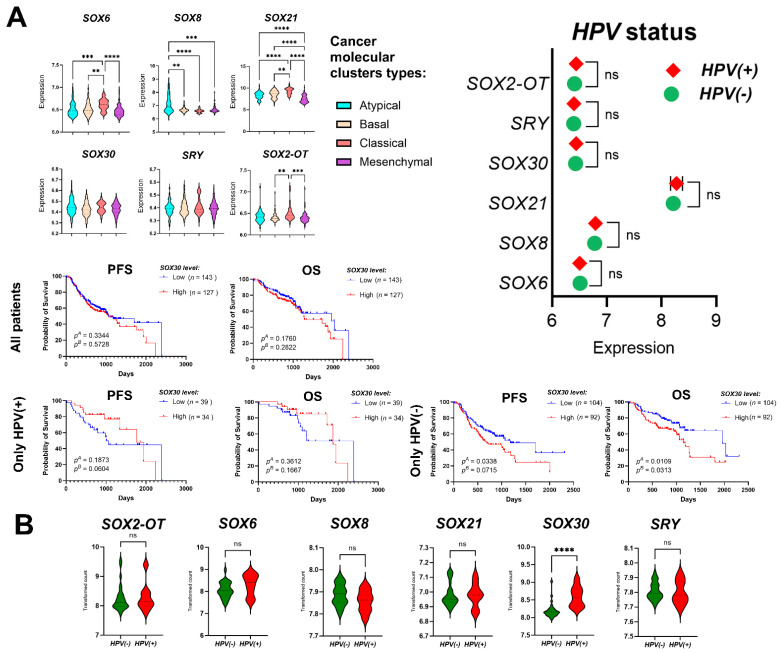
Validation of the results for *SRY*-related transcription factors using the GSE65858 and GSE3292 datasets. Expression level of *SRY*-related transcription factors depending on the viral status, molecular clusters and association between expression level of *SOX30* and patients’ OS and PFS in all patients, only in the HPV(+) and only in the HPV(−) groups of patients using the mean (Low vs. High) of gene expression level as cut-off based on GSE65858 dataset (**A**). Differences of *SRY*-related transcription factors expression levels depending on the viral status HPV(−) and HPV(+) based on GSE3292 dataset (**B**). *t*-test or Mann–Whitney U test, or ANOVA with Kruskal–Wallis post-test; *p^A^*—log-rank (Mantel–Cox) test; *p^B^*—Gehan–Breslow–Wilcoxon test; *p* < 0.05 considered as significant; ns: not significant, ** *p* < 0.01; *** *p* < 0.001; **** *p* < 0.0001.

Next, we decided to validate the TCGA data using the GSE65858 and GSE3292 GEO datasets [35,36]. First, we analyzed the GSE65858 dataset and analyzed only HPV(−) patients. We observed no significant differences (*p* > 0.05) in expression levels of all *SRY*-related transcription factors and gender, age, alcohol consumption, smoking status, cancer stage, T-stage or N-stage. Moreover, there were no differences (*p* > 0.05) in the case of HPV(+) and HPV(−) patients, nor in the case of viral activity, when the active (DNA+RNA+) and inactive infection (DNA+RNA-) status were compared, for all *SRY*-related transcription factors were indicated in the set of GSE65858 data. However, in the case of molecular clusters, we observed no differences between the atypical vs. basal vs. classical vs. mesenchymal subtypes in the case of *SOX30* and *SRY* (*p* = 0.1622 and *p* = 0.883, respectively). However, in the cases of the apical vs. basal subtypes for *SOX8* and atypical vs. classical for *SOX6*, *SOX8* and *SOX21*, as well as of the atypical vs. mesenchymal for *SOX8* and *SOX21,* significant differences were noticed (*p* = 0.0012; *p* = 0.0007, *p* < 0.0001 and *p* < 0.0001; *p* = 0.0003 and *p* < 0.0001, respectively). Surprisingly, we observed a similar pattern of expression in the case of *SOX2-OT*, *SOX6* and *SOX21* genes between the basal vs. classical as well as the classical vs. mesenchymal subtypes (*p* = 0.0018, *p* = 0.0033, *p* = 0.0094; and *p* = 0.0005, *p* < 0.0001, *p* < 0.0001; respectively). Only the expression level of *SOX21* was significantly higher in the basal than in the mesenchymal subtype of HNSCC (*p* < 0.0001). Next, we analyzed the association between high and low expression levels of *SRY*-related transcription factors and patients OS, and no significant differences between high and low expression levels were observed in the groups of all patients and only HPV(+). However, when expression levels of *SOX30* were compared in only HPV(−) groups of patients, we observed significantly longer PFS (*p* = 0.0338 and *p* = 0.0715) and OS for the patients with a lower expression level of *SOX30* than those with a higher expression of this gene (*p* = 0.0109 and *p* = 0.0313). All data are presented in Figure 7A and in Supplementary Table S4.

For the GSE329 dataset, we chose only HPV(−) patients and differences in expression levels of *SRY*-related transcription factors, depending on the clinicopathological parameters that were measured. We observed differences in the case of age (for *SOX30*: *p* = 0.0395), alcohol consumption (for *SOX30*: *p* = 0.0093; for *SRY*: *p* = 0.0102), pathological stage (for *SOX21*; *p* = 0.0064), pathological T-stage (for *SOX21*; *p* = 0.0005) and pathological N-stage (for *SOX21*; *p* = 0.0114 and for *SOX30*: *p* = 0.0114) and no differences in the expression levels of all *SRY*-related transcription factors for gender, tumor grade, clinical stage and clinical cervical lymph nodes status (*p* > 0.05). 

Next, the association between HPV status and expression levels of *SRY*-related transcription factors was analyzed. We observed that only for *SOX30* a significant up-regulation of expression level in the case of HPV+ patients (*p* < 0.0001) and no significant differences for *SOX2-OT*, *SOX6*, *SOX8*, *SOX21* and *SRY* genes (*p* > 0.05). All data are presented in Figure 7B and in Supplementary Table S5.

## 4. Discussion

The *SOX* gene family, also known as *SRY*-related HMG-box genes, constitutes the transcription factors which bind to the minor groove of DNA and regulate the transcription. In humans, 20 different protein-coding *SOX* genes have been described [37]. The *SOX* gene family plays a role in cancer development including important cancer-related processes such as epithelial–mesenchymal transition (EMT), the maintenance of cancer stem cell (CSC) population, metastasis and the regulation of drug resistance [38,39]. In this study, the selected *SRY*-related transcription factors, including lncRNA *SOX2-OT*, and protein-coding *SOX6*, *SOX8*, *SOX21*, *SOX30* and *SRY* genes were analyzed for the first time in HNSCC. Our results clearly point to *SOX6*, *SOX8* and *SRY* as factors which are characteristic for a tumor tissue. We correlated the expression levels of the *SOX* genes with a panel of clinicopathological parameters and observed the significant correlations between the gene expression and cancer site (oral cavity vs. pharynx or larynx). Moreover, we found a longer disease-free survival for patients with a higher *SOX21* expression and better survival rate for patients with a higher *SOX6* expression. Our study also showed the relevance of the analyzed genes with the immune response, as there were significant correlations between the expression of *SOX* genes and the estimate scores. 

*SOX2-OT* is the lncRNA *SOX2* overlapping transcript. Its gene consists of 10 exons and more than two transcription start sites. Some *SOX2-OT* lncRNA transcription variants can be up- or down-regulated in various cancer cell lines and tissues, since they are related to the cancer and the stem-cell-related pathways affecting tumor progression [40]. The *SOX2-OT* gene is placed within over 700 kb of a highly conserved region and is overexpressed in embryonic stem cells, and its expression decreases upon differentiation. In breast cancer, *SOX2-OT* takes part in the induction and/or maintenance of *SOX2* expression [38]. *SOX2* is a transcription factor responsible for maintaining the self-renewal and pluripotency of undifferentiated embryonic stem cells [21]. Its overexpression has been associated with tumorigenicity in HNSCC. *SOX2* drives the proliferation and expansion of tumor cells and inhibits their apoptosis. *SOX2* is described as the stem cell marker, and it has been detected in various cancer stem cell subpopulations including lung squamous cell carcinoma, medulloblastoma or CD44-positive prostate cancer [38,41]. We discovered that *SOX2-OT* expression is positively correlated with *SOX21* expression. In glioma, the increased *SOX21* expression inhibits *SOX2* leading to cancer cell apoptosis. A decreased *SOX2*/*SOX21* ratio results in the loss of stem cell features in glioma cells [42].

*SOX6* plays a role in cell growth, development and proliferation, especially during the development of the central nervous system, and the shaping of early embryonic muscle tissue and cartilage. *SOX6* can also be involved in tumorigenesis as an oncogene or tumor suppressor in different types of cancer [18]. It was demonstrated that transgenic mice with *SOX6* overexpression developed antitumor activity, caused by cytotoxic T cell activity. *SOX6* overexpression correlated with a longer survival in these mice compared to untreated mice [38]. The analyses of The Cancer Genome Atlas, Cancer Cell Line Encyclopedia and Genotype-Tissue Expression datasets, indicate that *SOX6* expression is decreased in breast cancer samples. Oppositely, *SOX6* overexpression inhibits the proliferation of cancer cells and promotes apoptosis. Moreover, methylation of the *SOX6* promoter is elevated when compared to non-cancerous tissues [43]. It has been demonstrated that *SOX6* expression is positively correlated with *SOX21* expression in glioma patients. Both *SOX6* and *SOX21* high-expression patients had a worse survival prognosis than those with a low expression of these genes [42].

*SOX8* is a transcription factor involved in the nervous system and mammalian testis development. It was identified as an oncogene, and it maintains the stemness in cancer stem cells [20]. In the study on *SOX8* in tongue squamous cell carcinoma, it was discovered that the knockout of this gene inhibited stem-like capacities, mesenchymal features and tumor metastasis in cancer chemoresistant cells. In tongue squamous cell carcinoma (TSCC), patients’ higher expression of *SOX8* caused lymph node metastasis, chemotherapeutic resistance and a poor prognosis [20]. Shuwei et al. conducted a study in which they identified *SOX8* as a regulator of a common oncogene—Golgi phosphoprotein 3 (*GOLPH3*) in TSCC. The knockdown of *SOX8* correlated with the suppression of *GOLPH3* promoter activity and the inhibition of TSCC cell proliferation in vivo and in vitro. Moreover, overexpressed *SOX8* was linked to *GOLPH3* promoter activation and elevated TSCC development. Additionally, *SOX8* was upregulated in tumor tissues in comparison to a non-cancerous control, which again correlated with *GOLPH3* levels [44]. In breast cancer patients, amplification of *SOX8* caused poor overall survival [45].

Another member of the *SRY*-related family, *SOX21,* is expressed in the anterior neural plate of mouse embryos and causes the progression of neurogenesis. The role of *SOX21* in human neural development is still unclear [14]. In glioma, *SOX21* is a tumor suppressor with a repression activity during tumorigenesis. It also reduces the stemness of tumor cells, leading to differentiation. The increased expression of *SOX21* in glioma cells inhibits the tumor progression and reduces the tumor size [38]. It is consistent with our results, as patients with a higher expression of *SOX21* have a longer disease-free survival. It can be assumed that a higher expression of *SOX21* reduces the number of cancer stem cells, resulting in a longer DFS.

*SOX30* participates in spermatogonial differentiation and the regulation of spermatogenesis. It can also function as a tumor suppressor in various cancers [46]. For example, in lung cancer, *SOX30* inhibits cell proliferation, migration, invasion, growth and tumor metastasis. The product of this gene stimulates p53 production, which promotes apoptosis [38]. Kumar et al. indicated that a lower expression of *SOX30* correlates with a poor prognosis and is associated with the malignant tumor type in bladder cancer [20]. Similarly, Yang et al. studied *SOX30* mRNA expression in bladder cancer and they discovered that it was lower in cancer cells than in noncancerous control tissues. Moreover, low *SOX30* expression correlated with reduced survival rates and its overexpression impaired cell proliferation, invasion and migration, and at the same time, it elevated the apoptosis of bladder cancer cells [46]. Another study indicated that the expression level of *SOX30* might be used as a prognostic biomarker in patients with ovarian cancer. Fai Han et al. demonstrated that a high *SOX30* expression is associated with a better overall survival in patients with the advanced-stage disease, but it is not associated with early stage patients. *SOX30* can also inhibit tumor metastasis by upregulating e-cadherin and downregulating n-catherin, vimentin and fibronectin and preventing epithelial-mesenchymal transmission (EMT) [47].

## 5. Conclusions

We demonstrated that the selected *SRY*-related transcription factors may act as prognostic biomarkers in HNSCC. In general, they show a lower expression in primary tumors than in normal tissue. Patients with a higher expression of these factors have higher OS and DFS rates, thus, a better prognosis. Moreover, *SOX30* seems to be a potential biomarker of HPV infection and could be used as a prognostic marker, but further research is required to fully understand the role of *SOX* family genes in HNSCC.

## Figures and Tables

**Table 1:** Differences in expression levels of SOX2-OT, SOX6, SOX8, SOX21, SOX30 and SRY genes depending on chosen clinical parameters. *t*-test or Mann–Whitney U test; *p* < 0.05 considered as significant.

Parameter	Group	*SOX2-OT*		*SOX6*		*SOX8*		*SOX21*		*SOX30*		*SRY*	
		Mean ± SEM	*p*-Value	Mean ± SEM	*p*-Value	Mean ± SEM	*p*-Value	Mean ± SEM	*p*-Value	Mean ± SEM	*p*-Value	Mean ± SEM	*p*-Value
Age	≤61	0.04783 ± 0.1198; *n* = 281	0.7991	−0.1924 ± 0.1048; *n* = 281	0.2216	−1.653 ± 0.1087; *n* = 281	0.2873	3.078 ± 0.1578; *n* = 281	0.3361	0.6262 ± 0.1301; *n* = 281	0.2568	0.8572 ± 0.08776; *n* = 281	0.04
	>61	0.1532 ± 0.1391; *n* = 240		−0.3326 ± 0.1070; *n* = 240		−1.885 ± 0.09462; *n* = 240		2.780 ± 0.1798; *n* = 240		0.3228 ± 0.1224; *n* = 240		0.6531 ± 0.09133; *n* = 240	
Gender	Female	0.04783 ± 0.1198; *n* = 281	0.7991	−0.1165 ± 0.1309; *n* = 137	0.4837	−1.964 ± 0.1156; *n* = 137	0.1797	2.373 ± 0.2214; *n* = 137	0.0013	0.1220 ± 0.1505; *n* = 137	0.0556	−0.3659 ± 0.005930; *n* = 137	<0.0001
	Male	0.1532 ± 0.1391; *n* = 240		−0.3071 ± 0.09033; *n* = 384		−1.687 ± 0.09008; *n* = 384		3.144 ± 0.1393; *n* = 384		0.6164 ± 0.1093; *n* = 384		1.166 ± 0.07612; *n* = 384	
Alcohol	Positive	0.1556 ± 0.1136; *n* = 348	0.5642	−0.2460 ± 0.09173; *n* = 348	0.7338	−1.713 ± 0.08719; *n* = 348	0.1459	2.857 ± 0.1488; *n* = 348	0.4252	0.5789 ± 0.1124; *n* = 348	0.2288	0.8373 ± 0.07675; *n* = 348	0.023
	Negative	0.002347 ± 0.1577; *n* = 163		−0.2776 ± 0.1351; *n* = 163		−1.856 ± 0.1399; *n* = 163		3.109 ± 0.1999; *n* = 163		0.2951 ± 0.1521; *n* = 163		0.6343 ± 0.1169; *n* = 163	
Smoking	Yes- and Ex-smoker	0.2712 ± 0.1096; *n* = 392	0.0023	−0.3185 ± 0.08872; *n* = 392	<0.0001	−1.751 ± 0.08042; *n* = 392	< 0.0001	3.082 ± 0.1368; *n* = 392	<0.0001	0.4488 ± 0.1002; *n* = 392	0.1163	0.8309 ± 0.07446; *n* = 392	0.0472
	Non-smoker	−0.4950 ± 0.1496; *n* = 117		−1.805 ± 0.1676; *n* = 117		2.525 ± 0.2500; *n* = 117		0.6635 ± 0.2126; *n* = 117		0.5933 ± 0.1253; *n* = 117		0.5933 ± 0.1253; *n* = 117	

**Table 2:** Differences in expression levels of SOX2-OT, SOX6, SOX8, SOX21, SOX30 and SRY genes depending on chosen pathological parameters. *t*-test or Mann–Whitney U test; *p* < 0.05 considered as significant.

Parameter	Group	*SOX2-OT*		*SOX6*		*SOX8*		*SOX21*		*SOX30*		*SRY*	
		Mean ± SEM	*p*-Value	Mean ± SEM	*p*-Value	Mean ± SEM	*p*-Value	Mean ± SEM	*p*-Value	Mean ± SEM	*p*-Value	Mean ± SEM	*p*-Value
Cancer Stage	I + II	0.2040 ± 0.2092; *n* = 117	0.924	0.1695 ± 0.1433; *n* = 117	0.0014	−1.904 ± 0.1304; *n* = 117	0.5406	2.957 ± 0.2369; *n* = 117	0.7	0.2561 ± 0.1733; *n* = 117	0.2227	0.7084 ± 0.1211; *n* = 117	0.9845
	III + IV	0.06481 ± 0.1033; *n* = 390		−0.3372 ± 0.08720; *n* = 390		−1.700 ± 0.08909; *n* = 390		2.964 ± 0.1389; *n* = 390		0.5903 ± 0.1072; *n* = 390		0.7934 ± 0.07595; *n* = 390	
T Stage	T1 + T2	0.2248 ± 0.2679; *n* = 49	0.5677	−0.01565 ± 0.2695; *n* = 49	0.1375	−1.401 ± 0.2418; *n* = 49	0.0733	3.178 ± 0.3608; *n* = 49	0.3988	0.4485 ± 0.3039; *n* = 49	0.8232	0.9239 ± 0.2115; *n* = 49	0.2598
	T3 + T4	0.04335 ± 0.1013; *n* = 410		−0.3313 ± 0.08443; *n* = 410		−1.861 ± 0.07732; *n* = 410		2.832 ± 0.1321; *n* = 410		0.2970 ± 0.09282; *n* = 410		0.6437 ± 0.06757; *n* = 410	
N Stage	N0 + N1	−0.1296 ± 0.1123; *n* = 242	0.2572	−0.2025 ± 0.1081; *n* = 242	0.1625	−1.845 ± 0.1005; *n* = 242	0.1763	2.988 ± 0.1669; *n* = 242	0.5351	0.05867 ± 0.1205; *n* = 242	0.0008	0.7524 ± 0.09294; *n* = 242	0.4682
	N2 + N3	0.2947 ± 0.1767; *n* = 179		−0.4931 ± 0.1364; *n* = 179		−1.685 ± 0.1211; *n* = 179		2.815 ± 0.2017; *n* = 179		0.5726 ± 0.1409; *n* = 179		0.6242 ± 0.09903; *n* = 179	
Grade	G1 + G2	0.01121 ± 0.1074; *n* = 368	0.2242	−0.2435 ± 0.08779; *n* = 368	0.3276	−1.940 ± 0.07623; *n* = 368	0.0004	2.958 ± 0.1342; *n* = 368	0.8503	0.1451 ± 0.09247; *n* = 368	<0.0001	0.6729 ± 0.07275; *n* = 368	0.1391
	G3 + G4	0.1540 ± 0.1750; *n* = 131		−0.4276 ± 0.1544; *n* = 131		−1.361 ± 0.1612; *n* = 131		2.765 ± 0.2660; *n* = 131		1.177 ± 0.2132; *n* = 131		0.9374 ± 0.1368; *n* = 131	
Perineural invasion	Positive	−0.3446 ± 0.1501; *n* = 169	0.0003	−0.4608 ± 0.1270; *n* = 169	0.2902	−1.985 ± 0.08808; *n* = 169	0.7462	2.222 ± 0.1991; *n* = 169	0.0007	0.1076 ± 0.1265; *n* = 169	0.321	0.5787 ± 0.09613; *n* = 169	0.6863
	Negative	0.3188 ± 0.1490; *n* = 195		−0.3495 ± 0.1333; *n* = 195		−1.720 ± 0.1292; *n* = 195		3.116 ± 0.1936; *n* = 195		0.3887 ± 0.1430; *n* = 195		0.5730 ± 0.09789; *n* = 195	
Lymph node neck dissection	Positive	−0.01959 ± 0.1016; *n* = 421	0.0018	−0.3538 ± 0.08515; *n* = 421	0.0165	−1.811 ± 0.07666; *n* = 421	0.4513	2.774 ± 0.1316; *n* = 421	0.0019	0.2630 ± 0.09197; *n* = 421	<0.0001	0.7351 ± 0.06894; *n* = 421	0.6681
	Negative	0.6196 ± 0.2022; *n* = 97		0.1205 ± 0.1519; *n* = 97		−1.527 ± 0.2069; *n* = 97		3.620 ± 0.2725; *n* = 97		1.505 ± 0.2486; *n* = 97		0.9131 ± 0.1620; *n* = 97	
Lymphovascular invasion	Positive	0.4095 ± 0.2077; *n* = 125	0.0242	−0.4722 ± 0.1745; *n* = 125	0.7387	−1.706 ± 0.1411; *n* = 125	0.0449	3.139 ± 0.2387; *n* = 125	0.0371	0.5804 ± 0.1807; *n* = 125	0.0089	0.6793 ± 0.1221; *n* = 125	0.2787
	Negative	−0.2250 ± 0.1262; *n* = 225		−0.3567 ± 0.1143; *n* = 225		−1.973 ± 0.09506; *n* = 225		2.538 ± 0.1816; *n* = 225		0.02504 ± 0.1149; *n* = 225		0.5405 ± 0.08737; *n* = 225	
HPV in p16 test	Positive	1.157 ± 0.2662; *n* = 39	0.009	−0.1204 ± 0.3127; *n* = 39	0.2585	−0.5596 ± 0.4108; *n* = 39	0.0147	4.145 ± 0.3795; *n* = 39	0.0559	3.471 ± 0.3312; *n* = 39	<0.0001	1.423 ± 0.2762; *n* = 39	0.1264
	Negative	0.3251 ± 0.2632; *n* = 73		−0.3236 ± 0.2012; *n* = 73		−1.633 ± 0.1988; *n* = 73		3.295 ± 0.2976; *n* = 73		−0.1825 ± 0.1713; *n* = 73		0.8632 ± 0.1700; *n* = 73	

## Data Availability

The datasets used and/or analyzed during the current study are available from the corresponding author on reasonable request. Raw data are available from the XenaBrowser, Ualcan, cBioportal, GEPIA2 and ESTIMATE databases.

## Supplementary Materials

The following supporting information can be downloaded at: https://www.mdpi.com/article/10.3390/cimb45120592/s1, Supplementary Table S1: Positive and negative correlation of *SOX2-OT*, *SOX6*, *SOX8*, *SOX21* and *SOX30* with genes involved in the important cellular processes. Only genes with Spearman’s correlation R > 0.3, R < –0.3 and *p* < 0.05 were indicated in REACTOME pathway analysis; Supplementary Table S2: Differences between patients with low and high expression of *SRY*-related genes in context of their stromal score, immune score and estimate score; *t*-test or Mann–Whitney U test; *p* < 0.05 considered as significant; Supplementary Table S3: Immunological profile of HSNCC patients depending on the expression levels of *SOX2-OT*, *SOX8*, *SOX21* and *SOX30*. Differences in the population of lymphocytes, neutrophils, eosinophils, mast cells, dendritic cells and macrophages (A), and in the specific subpopulation of B cells, macrophages and T cells (B). *t*-test or Mann–Whitney U test; *p* < 0.05 considered as significant; Supplementary Table S4: Validation of the results for *SRY*-related transcription factors using the GSE65858 dataset; Supplementary Table S5: Validation of the results for *SRY*-related transcription factors using the GSE65858 dataset.

## References

[1] Marur S., Forastiere A.A. (2008). Head and neck cancer: Changing epidemiology, diagnosis, and treatment. Mayo Clin. Proc..

[2] Dyzmann-Sroka A., Malicki J., Jędrzejczak A. (2020). Cancer incidence in the Greater Poland region as compared to Europe. Rep. Pract. Oncol. Radiother. J. Greatpoland Cancer Cent. Pozn. Pol. Soc. Radiat. Oncol..

[3] Leemans C.R., Braakhuis B.J., Brakenhoff R.H. (2011). The molecular biology of head and neck cancer. Nat. Rev. Cancer.

[4] Cancer Genome Atlas Network (2015). Comprehensive genomic characterization of head and neck squamous cell carcinomas. Nature.

[5] Dok R., Nuyts S. (2016). HPV Positive Head and Neck Cancers: Molecular Pathogenesis and Evolving Treatment Strategies. Cancers.

[6] Alsahafi E., Begg K., Amelio I., Raulf N., Lucarelli P., Sauter T., Tavassoli M. (2019). Clinical update on head and neck cancer: Molecular biology and ongoing challenges. Cell Death Dis..

[7] Rupar M.J., Golusinski P., Golusinski W., Masternak M.M. (2019). Human Papillomavirus and the use of nanoparticles for immunotherapy in HPV-related cancer: A review. Rep. Pract. Oncol. Radiother. J. Greatpoland Cancer Cent. Pozn. Pol. Soc. Radiat. Oncol..

[8] Rajabi-Moghaddam M., Abbaszadeh H. (2022). Gene polymorphisms and prognosis of head and neck squamous cell carcinoma: A systematic review. Rep. Pract. Oncol. Radiother. J. Greatpoland Cancer Cent. Pozn. Pol. Soc. Radiat. Oncol..

[9] Rajabi-Moghaddam M., Abbaszadeh H. (2022). Gene polymorphisms and risk of head and neck squamous cell carcinoma: A systematic review. Rep. Pract. Oncol. Radiother. J. Greatpoland Cancer Cent. Pozn. Pol. Soc. Radiat. Oncol..

[10] Guglas K., Kozłowska-Masłoń J., Kolenda T., Paszkowska A., Teresiak A., Bliźniak R., Lamperska K. (2022). Midsize noncoding RNAs in cancers: A new division that clarifies the world of noncoding RNA or an unnecessary chaos?. Rep. Pract. Oncol. Radiother. J. Greatpoland Cancer Cent. Pozn. Pol. Soc. Radiat. Oncol..

[11] Kolenda T., Guglas K., Ryś M., Bogaczyńska M., Teresiak A., Bliźniak R., Łasińska I., Mackiewicz J., Lamperska K.M. (2017). Biological role of long non-coding RNA in head and neck cancers. Rep. Pract. Oncol. Radiother. J. Greatpoland Cancer Cent. Pozn. Pol. Soc. Radiat. Oncol..

[12] Kolenda T., Guglas K., Kopczyńska M., Sobocińska J., Teresiak A., Bliźniak R., Lamperska K. (2020). Good or not good: Role of miR-18a in cancer biology. Rep. Pract. Oncol. Radiother. J. Greatpoland Cancer Cent. Pozn. Pol. Soc. Radiat. Oncol..

[13] Kolenda T., Paszkowska A., Braska A., Kozłowska-Masłoń J., Guglas K., Poter P., Wojtczak P., Bliźniak R., Lamperska K., Teresiak A. (2023). Host gene and its guest: Short story about relation of long-noncoding *MIR31HG* transcript and microRNA *miR-31*. Rep. Pract. Oncol. Radiother. J. Greatpoland Cancer Cent. Pozn. Pol. Soc. Radiat. Oncol..

[14] Kolenda T., Kopczyńska M., Guglas K., Teresiak A., Bliźniak R., Łasińska I., Mackiewicz J., Lamperska K. (2018). EGOT lncRNA in head and neck squamous cell carcinomas. Pol. J. Pathol. Off. J. Pol. Soc. Pathol..

[15] Kopczyńska M., Kolenda T., Guglas K., Sobocińska J., Teresiak A., Bliźniak R., Mackiewicz A., Mackiewicz J., Lamperska K. (2020). PRINS lncRNA Is a New Biomarker Candidate for HPV Infection and Prognosis of Head and Neck Squamous Cell Carcinomas. Diagnostics.

[16] Kolenda T., Guglas K., Baranowski D., Sobocińska J., Kopczyńska M., Teresiak A., Bliźniak R., Lamperska K. (2020). cfRNAs as biomarkers in oncology—Still experimental or applied tool for personalized medicine already?. Rep. Pract. Oncol. Radiother. J. Greatpoland Cancer Cent. Pozn. Pol. Soc. Radiat. Oncol..

[17] Kozłowska J., Kolenda T., Poter P., Sobocińska J., Guglas K., Stasiak M., Bliźniak R., Teresiak A., Lamperska K. (2021). Long Intergenic Non-Coding RNAs in HNSCC: From “Junk DNA” to Important Prognostic Factor. Cancers.

[18] Lv L., Zhou M., Zhang J., Liu F., Qi L., Zhang S., Bi Y., Yu Y. (2020). SOX6 suppresses the development of lung adenocarcinoma by regulating expression of p53, p21CIPI, cyclin D1 and β-catenin. FEBS Open Bio.

[19] Liang Z., Xu J., Gu C. (2020). Novel role of the SRY-related high-mobility-group box D gene in cancer. Semin. Cancer Biol..

[20] Xie S.L., Fan S., Zhang S.Y., Chen W.X., Li Q.X., Pan G.K., Zhang H.Q., Wang W.W., Weng B., Zhang Z. (2018). SOX8 regulates cancer stem-like properties and cisplatin-induced EMT in tongue squamous cell carcinoma by acting on the Wnt/β-catenin pathway. Int. J. Cancer.

[21] Shahryari A., Jazi M.S., Samaei N.M., Mowla S.J. (2015). Long non-coding RNA SOX2OT: Expression signature, splicing patterns, and emerging roles in pluripotency and tumorigenesis. Front. Genet..

[22] Staniewska E., Tomasik B., Tarnawski R., Łaszczych M., Miszczyk M. (2021). The prognostic value of red cell distribution width (RDW), neutrophil-to-lymphocyte ratio (NLR), and platelet-to-lymphocyte ratio (PLR) in radiotherapy for oropharyngeal cancer. Rep. Pract. Oncol. Radiother. J. Greatpoland Cancer Cent. Pozn. Pol. Soc. Radiat. Oncol..

[23] Ganesh M.S., Narayanan G.S., Kumar R. (2020). Change of telomerase activity in peripheral blood of patients with head and neck squamous cell carcinoma pre and post curative treatment. Rep. Pract. Oncol. Radiother. J. Greatpoland Cancer Cent. Pozn. Pol. Soc. Radiat. Oncol..

[24] Pietrzak A.K., Kazmierska J., Marszalek A., Golusinski P., Heydrych A., Wiechec K., Cholewinski W. (2020). Dual-time-point PET/CT study protocol can improve the larynx cancer diagnosis. Rep. Pract. Oncol. Radiother. J. Greatpoland Cancer Cent. Pozn. Pol. Soc. Radiat. Oncol..

[25] Sindhu S.K., Bauman J.E. (2019). Current Concepts in Chemotherapy for Head and Neck Cancer. Oral Maxillofac. Surg. Clin. N. Am..

[26] Gordon K., Gulidov I., Semenov A., Golovanova O., Koryakin S., Makeenkova T., Ivanov S., Kaprin A. (2021). Proton re-irradiation of unresectable recurrent head and neck cancers. Rep. Pract. Oncol. Radiother. J. Greatpoland Cancer Cent. Pozn. Pol. Soc. Radiat. Oncol..

[27] Koiwai K., Hirasawa D., Sugimura M., Endo Y., Mizuhata K., Ina H., Fukazawa A., Kitoh R., Sakai H., Fujinaga Y. (2022). Impact of upgraded radiotherapy system on outcomes in postoperative head and neck squamous cell carcinoma patients. Rep. Pract. Oncol. Radiother. J. Greatpoland Cancer Cent. Pozn. Pol. Soc. Radiat. Oncol..

[28] Viani G.A., Faustino A.C., Danelichen AF B., Matsuura F.K., Neves LV F., Fernandes M.H., Fernandes J.P. (2021). Radiotherapy for locally advanced head and neck cancer in elderly patients: Results and prognostic factors a single cohort. Rep. Pract. Oncol. Radiother. J. Greatpoland Cancer Cent. Pozn. Pol. Soc. Radiat. Oncol..

[29] Chandrashekar D.S., Karthikeyan S.K., Korla P.K., Patel H., Shovon A.R., Athar M., Netto G.J., Qin Z.S., Kumar S., Manne U. (2022). UALCAN: An update to the integrated cancer data analysis platform. Neoplasia.

[30] Sobocińska J., Nowakowska J., Molenda S., Olechnowicz A., Guglas K., Kozłowska-Masłoń J., Kazimierczak U., Machnik M., Oleksiewicz U., Teresiak A. (2022). Zinc Finger Proteins in Head and Neck Squamous Cell Carcinomas: ZNF540 May Serve as a Biomarker. Curr. Oncol. (Tor. Ont.).

[31] Tomaszewska W., Kozłowska-Masłoń J., Baranowski D., Perkowska A., Szałkowska S., Kazimierczak U., Severino P., Lamperska K., Kolenda T. (2021). *miR-154* Influences HNSCC Development and Progression through Regulation of the Epithelial-to-Mesenchymal Transition Process and Could Be Used as a Potential Biomarker. Biomedicines.

[32] Koteluk O., Bielicka A., Lemańska Ż., Jóźwiak K., Klawiter W., Mackiewicz A., Kazimierczak U., Kolenda T. (2021). The Landscape of Transmembrane Protein Family Members in Head and Neck Cancers: Their Biological Role and Diagnostic Utility. Cancers.

[33] Paszkowska A., Kolenda T., Guglas K., Kozłowska-Masłoń J., Podralska M., Teresiak A., Bliźniak R., Dzikiewicz-Krawczyk A., Lamperska K. (2022). C10orf55, CASC2, and SFTA1P lncRNAs Are Potential Biomarkers to Assess Radiation Therapy Response in Head and Neck Cancers. J. Pers. Med..

[34] Kolenda T., Poter P., Guglas K., Kozłowska-Masłoń J., Braska A., Kazimierczak U., Teresiak A. (2023). Biological role and diagnostic utility of ribosomal protein L23a pseudogene 53 in cutaneous melanoma. Rep. Pract. Oncol. Radiother. J. Greatpoland Cancer Cent. Pozn. Pol. Soc. Radiat. Oncol..

[35] Wichmann G., Rosolowski M., Krohn K., Kreuz M., Boehm A., Reiche A., Scharrer U., Halama D., Bertolini J., Bauer U. (2015). The role of HPV RNA transcription, immune response-related gene expression and disruptive TP53 mutations in diagnostic and prognostic profiling of head and neck cancer. Int. J. Cancer.

[36] Wang K.P., Yuan Y.J., Zhu J.Q., Li B.L., Zhang T.T. (2020). Analysis of key genes and signal pathways of human papilloma virus-related head and neck squamous cell carcinoma. Chin. J. Stomatol..

[37] Bowles J., Schepers G., Koopman P. (2000). Phylogeny of the SOX family of developmental transcription factors based on sequence and structural indicators. Dev. Biol..

[38] Grimm D., Bauer J., Wise P., Krüger M., Simonsen U., Wehland M., Infanger M., Corydon T.J. (2020). The role of SOX family members in solid tumours and metastasis. Semin. Cancer Biol..

[39] Pouremamali F., Vahedian V., Hassani N., Mirzaei S., Pouremamali A., Kazemzadeh H., Faridvand Y., Jafari-Gharabaghlou D., Nouri M., Maroufi N.F. (2022). The role of SOX family in cancer stem cell maintenance: With a focus on SOX2. Pathology.

[40] Wang Y., Wu N., Luo X., Zhang X., Liao Q., Wang J. (2020). SOX2OT, a novel tumor-related long non-coding RNA. Biomed. Pharmacother. = Biomed. Pharmacother..

[41] Kumar P., Mistri T.K. (2020). Transcription factors in SOX family: Potent regulators for cancer initiation and development in the human body. Semin. Cancer Biol..

[42] Jiang L., Yang H., Chen T., Zhu X., Ye J., Lv K. (2020). Identification of HMG-box family establishes the significance of SOX6 in the malignant progression of glioblastoma. Aging.

[43] Zhang L., Niu X., Zhang X., Zhan G., Xue X., Wang X., Zhang H., Guo Z. (2021). SRY-related high-mobility-group box 6 suppresses cell proliferation and is downregulated in breast cancer. Anti-Cancer Drugs.

[44] Chen S., Li H., Li X., Chen W., Zhang X., Yang Z., Chen Z., Chen J., Zhang Y., Shi D. (2020). High SOX8 expression promotes tumor growth and predicts poor prognosis through GOLPH3 signaling in tongue squamous cell carcinoma. Cancer Med..

[45] Mehta G.A., Khanna P., Gatza M.L. (2019). Emerging Role of SOX Proteins in Breast Cancer Development and Maintenance. J. Mammary Gland Biol. Neoplasia.

[46] Liu C., Liu Y., Tian J., Zhang S., Li X., Zhai X., Feng Q. (2020). High expression of SRY-box transcription factor 30 associates with well differentiation, absent lymph node metastasis and predicts longer survival in nonsmall-cell lung cancer patients. Medicine.

[47] Han F., Liu W.B., Li J.J., Zhang M.Q., Yang J.T., Zhang X., Hao X.L., Yin L., Mao C.Y., Jiang X. (2019). SOX30 is a prognostic biomarker and chemotherapeutic indicator for advanced-stage ovarian cancer. Endocr.-Relat. Cancer.

